# Decoverslipping of Archival Cytology Aspirate Smears for Ancillary Studies: A Review of Methods and Institutional Experience With Freezer‐Based Techniques

**DOI:** 10.1111/cyt.70097

**Published:** 2026-06-05

**Authors:** Nyein Nyein Htun, Sinchita Roy‐Chowdhuri, Benjamin B. Morris, Gloria Hopkins Sura

**Affiliations:** ^1^ Department of Anatomic Pathology, Division of Pathology and Laboratory Medicine The University of Texas MD Anderson Cancer Center Houston Texas USA; ^2^ Department of Thoracic/Head and Neck Medical Oncology The University of Texas MD Anderson Cancer Center Houston Texas USA

**Keywords:** ancillary testing, aspirate smear, biomarker, coverslip, cytology, decoverslipping, molecular testing, mounting medium, preanalytical

## Abstract

**Introduction:**

Archival cytology aspirate smears are increasingly used for ancillary testing, yet decoverslipping remains a practical bottleneck. This study reviews published decoverslipping methodologies and evaluates freezer‐based strategies for aspirate smears.

**Methods:**

Published decoverslipping techniques were reviewed and compared with respect to turnaround time, tissue preservation, safety and suitability for downstream applications. An institutional study at MD Anderson Cancer Center evaluated freezer‐based decoverslipping (−20°C and −80°C) of archival aspirate smears (40 cases, 80 slides) across four freeze‐duration groups (1–2 min, 3–5 min, 10 min and 15 min).

**Results:**

Aspirate smears prepared with thick xylene‐based mounting media and rigid glass coverslips were consistently resistant to cold‐based removal, with complete removal achieved in only 26 of 80 slides (32.5%), corresponding to 22 of 76 evaluable aspirate slides (28.9%). The highest per‐slide success rate was observed at 10 min (15/40; 37.5%). A directional advantage for −80°C over −20°C was apparent but insufficient to overcome the fundamental resistance of this preparation type. Marked intra‐case variability indicated that slide‐specific factors were the dominant determinants of outcome.

**Conclusion:**

Mounting‐media thickness and coverslip composition are primary determinants of decoverslipping success and represent under‐recognised preanalytical variables. These findings underscore the need to align coverslipping workflows with anticipated downstream ancillary testing requirements.

## Graphical abstract

Freezer‐based decoverslipping of archival cytology aspirate smears achieved only 32.5% slide‐level success (26/80), with mounting medium composition and coverslip material, not freezing temperature or duration, identified as dominant determinants, underscoring the need to align coverslipping practices with anticipated ancillary testing requirements.

Archival cytology aspirate smears are increasingly needed for downstream ancillary testing, yet removing the coverslip remains a practical bottleneck. This study reviews published decoverslipping methods and reports institutional experience at MD Anderson Cancer Center showing that freezer‐based techniques achieved complete coverslip removal in only 28.9% of aspirate smear slides, with mounting‐medium composition and coverslip material, not freezing protocol, identified as the dominant determinants of success. Practical recommendations for prospectively aligning coverslipping practices with ancillary testing needs are provided.

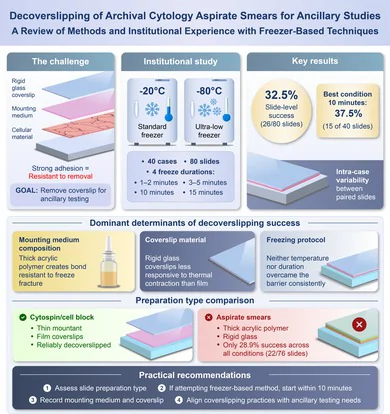

## 1 Introduction

Archival cytology aspirate smears are increasingly used for ancillary studies, including immunohistochemistry, fluorescence in situ hybridisation and high‐throughput molecular assays [1, 2]. Accessing cellular material from permanently mounted slides requires removal of the coverslip, a process termed decoverslipping. Traditionally, decoverslipping is performed by prolonged immersion in xylene until the mounting medium dissolves. Although reliable, this approach may require hours to days, involves hazardous solvent exposure, and is suboptimal for preserving nucleic acids [1, 2].

With the expanding role of biomarkers as ancillary diagnostics, particularly assays requiring protein preservation or high‐quality deoxyribonucleic acid (DNA) or ribonucleic acid (RNA), there is renewed interest in faster, safer and decoverslipping techniques that preserve both morphology and nucleic acid integrity [1, 2, 3, 4]. Numerous alternative methods have been described, including freezing‐based approaches, heated solvents, mechanical techniques, ultrasonic vibration and hybrid thermal–mechanical strategies [5, 6, 7, 8, 9, 10, 11, 12, 13, 14]. However, most published studies focus on histology sections or cytology preparations with variable coverslip and mounting‐media characteristics [1, 2, 3, 4].

This manuscript provides (1) a comprehensive review of decoverslipping methodologies reported in the literature, summarised in Tables 1 and 2, and (2) an institutional study highlighting the unique challenges encountered when decoverslipping aspirate cytology smears for planned downstream ancillary testing. These data emphasise the importance of coverslip composition and mounting‐media selection as critical preanalytical determinants of ancillary testing feasibility.

**TABLE 1:** Summary of published decoverslipping methods and outcomes.

Study	Mounting medium	Coversli*p*	Decoverslipping method	Analysis	Key findings
Dejmek et al. [6] Cancer Cytopathol 2013	EcoMount (xylene‐free) Pertex (xylene‐based)	NS	Histo‐Clear for EcoMount Xylene for Pertex	PCR	EcoMount/Histo‐Clear yielded significantly higher DNA quantity and quality than Pertex/xylene.
Zhou et al. [7] J Am Soc Cytopathol 2017	Toluene‐based (Richard‐Allan Scientific)	Glass	1. LN_2_ (30 s) ± razor blade + 15‐min xylene rinse 2. Xylene immersion (3–4 days)	RT‐PCR; real‐time PCR	LN_2_ method produced equivalent DNA/RNA yield and amplifiability to multi‐day xylene; no compromise in nucleic acid integrity.
da Cunha Santos et al. [12] Cancer Cytopathol 2013	NS	NS	‘Freezer method’: −20°C for 1–2 min; coverslip removed with scalpel.	NS	Letter describing the freezer method as a rapid alternative to xylene soaking for DNA retrieval.
Ramesh et al. [5] World J Methodol 2019	NS	NS	−80°C for 2 h after scoring edges; razor blade removal. 20‐min xylene soak if adherent. Note: FFPE sections; deparaffinisation, destaining, ethanol rehydration.	PCR	Xylene yielded higher‐quality DNA; −80°C freezing workable but not superior; often required supplemental xylene.
Dinesh et al. [9] J Pharm Negative Results 2022	DPX	Glass	1. Xylene (RT) 2. Heated xylene (56°C) 3. Acetone (~56 h) 4. Freezer (−18°C) 5. Endo‐Frost spray	IHC; decoverslipping time	Freezing methods (Endo‐Frost, −18°C) fastest and most effective; good‐quality IHC on archival H&E slides.
Karigoudar et al. [10] J Clin Diagn Res 2019	DPX	Glass	1. Freezing (0°C to −4°C) 2. Xylene (RT) 3. Heated xylene (56°C) 4. Petrol 5. Diesel (ineffective)	Decoverslipping time	Freezing most efficient (days → minutes). Heated xylene and petrol moderately improved. Diesel ineffective.
Cozma and Henwood [8] J Histotechnol 2019	NS	Glass	1. LN_2_ (30 s) + microtome blade + xylene 2. Ultrasonic vibration in xylene 3. −80°C (5–15 min) + manual removal + xylene	Time; feasibility	–80°C (5–10 min) safe, effective, dramatically faster. LN_2_ and ultrasonic methods ineffective (cracking, tissue loss).
Sabarinath et al. [11] J Oral Maxillofac Pathol 2023	DPX	Glass	Dry ice (−78.5°C, 15–60 s); microtome blade; 2‐min xylene rinse.	Removal efficiency; tissue integrity; H&E restaining	Rapid (30–40 s), inexpensive; preserved morphology and staining quality with minimal xylene exposure.
Millar and Unsicker [13] J Neurosci Methods 1983	Araldite epoxy resin	Collagen‐coated glass	Coverslips scored, water applied, ice block rubbed on surface. Thermal expansion separated glass from resin.	Cell preservation; EM relocation	Simple, nondamaging for epoxy‐embedded neuronal cultures. Not applicable to standard histology/cytology slides with DPX.
Pedraza and Marciano [14] J Forensic Sci 2025	Eukitt; Cytoseal	Glass	Humid chambSer (30°C–37°C, 6–7 days); nail polish on surface; edges scored and coverslip lifted. Difficult cases: 2–24 h additional incubation.	NS	100% success (35/35 slides, 1 week–6 years). Nondestructive, chemical‐free; minor cracking mitigated by longer incubation.

Abbreviations: DPX, Distrene–Plasticiser–Xylene; EM, electron microscopy; FFPE, formalin‐fixed paraffin‐embedded; H&E, haematoxylin and eosin; IHC, immunohistochemistry; LN_2_, liquid nitrogen; NS, not specified; PCR, polymerase chain reaction; RT, room temperature; RT‐PCR, reverse‐transcription PCR.

**TABLE 2:** Summary of published decoverslipping methods.

Method	Turnaround	Key advantages	Key limitations	References
Xylene soaking (room temperature)	1 h–4 days	Simple, low cost, broadly effective	Prolonged; toxic solvent exposure; suboptimal for nucleic acid preservation	[4, 5, 6, 7, 8, 9, 10]
Dry ice (−78.5°C)	15–60 s	Rapid; low cost; reduced solvent exposure	Requires careful handling; precise timing needed	[11]
Freezer (−18°C to −20°C)	1–10 min	Simple; widely accessible; no specialised equipment	Variable effectiveness; often requires supplemental xylene	[9, 10, 12]
Ultra‐low freezer (−80°C)	5–15 min	Relatively rapid; preserves morphology	Requires specialised equipment; thick mountants may resist	[5, 8]
Liquid nitrogen (−196°C)	~30 s	Extremely rapid; preserves DNA/RNA	High risk of glass cracking; specialised safety infrastructure required	[8]
Cryogenic spray	~5 min	Rapid surface cooling	Variable performance; risk of brittle fracture	[9, 10]
Heated xylene (56°C)	< 15 min	Faster than room temperature xylene	Solvent exposure persists; potential heat‐related analyte degradation	[9]
Hot plate (120°C–170°C)	< 2 min	Extremely rapid	DNA/RNA fragmentation risk; may limit molecular applications	[4]
Ultrasonic vibration	Variable	Agitation loosens coverslip	Slow; tissue loss; slide cracking	[8]

## 2 Review of Decoverslipping Methods

### 2.1 Traditional Xylene Soaking

Xylene dissolution remains the most widely utilised decoverslipping method. Slides are immersed in xylene at room temperature until the mounting medium dissolves. Turnaround time ranges from 1 h to 4 days, depending on slide age, mountant composition and thickness. The method is valued for its simplicity, low cost and broad effectiveness [5, 6, 7, 8, 9, 10] but is limited by prolonged processing, significant toxic solvent exposure and environmental disposal concerns. It is frequently used as the reference standard against which alternative techniques are evaluated [5, 6, 7, 8, 9, 10].

### 2.2 Dry Ice Freezing (−78.5°C)

Slides are placed coverslip‐side down on dry ice for 15–60 s, embrittling the mounting medium and allowing the coverslip to be lifted with a microtome blade, followed by a brief xylene rinse [11]. Advantages include rapid turnaround, low cost, morphological preservation and reduced xylene exposure; limitations relate to careful handling and precise timing to avoid slide damage [11].

### 2.3 Freezer Method (−18°C to −20°C)

Slides are placed in a standard laboratory freezer for 1–2 min to embrittle the mounting medium, after which the coverslip is removed mechanically [9, 12]. Freezing at 0°C to −4°C for approximately 10 min has also been described [10]. These approaches are simple and widely accessible, but variably effective and supplemental xylene treatment is often necessary.

### 2.4 Ultra‐Low Freezer Method (−80°C)

Slides are cooled at −80°C for 5–15 min, or up to 2 h, prior to mechanical coverslip removal [5, 8]. This approach preserves tissue morphology and is effective for many histology and cytology slide types, though it requires specialised equipment and may still necessitate supplemental xylene for thickly mounted slides.

### 2.5 Liquid Nitrogen Freezing (−196°C)

Slides are immersed in liquid nitrogen for approximately 30 s, followed by mechanical removal and a brief xylene rinse [8]. This technique preserves both DNA and RNA integrity but carries a high risk of glass cracking and tissue loss and requires specialised safety infrastructure, making it impractical for routine diagnostic use [8].

### 2.6 Aerosol Cryogenic Sprays

Cryogenic sprays such as Endo‐Frost rapidly cool the coverslip surface to facilitate detachment [5, 6]. Removal has been reported in as few as 5 min; however, performance is variable and rapid cooling may cause brittle glass fracture [9, 10].

### 2.7 Heated Xylene

Slides are immersed in xylene warmed to approximately 56°C, reducing decoverslipping time to under 15 min [9]. However, organic solvent exposure is not eliminated, and heat‐related degradation of molecular analytes is a concern.

### 2.8 Hot‐Plate Method

Slides are placed on a heated surface (120°C for up to 30 s for fresh slides; up to 170°C for 2 min for older slides) to soften the mounting medium prior to mechanical removal [13]. Decoverslipping is rapid, but the high temperatures raise concerns regarding protein and nucleic acid degradation that may limit suitability for downstream testing.

### 2.9 Ultrasonic Vibration

Slides submerged in xylene are placed in an ultrasonic bath to loosen the coverslip [8]. This method is slow, labour‐intensive and associated with tissue loss and slide cracking.

### 2.10 Mechanical/Thermal Hybrid Techniques

The ice‐block and scored‐coverslip method, originally described for neuronal cultures in epoxy resin [10], relies on thermal expansion of water to separate the coverslip. It is unreliable for routine histology or cytology slides mounted with DPX (distyrene, plasticiser and xylene) or similar media and is associated with brittle fracture and tissue loss [13].

## 3 MD Anderson Cancer Center Experience: Freezing Methods of Decoverslipping Fine Needle Aspirate Smears

### 3.1 Methods

#### 3.1.1 Study Material

Archival, de‐identified cytology slides from clinical cases accessioned at The University of Texas MD Anderson Cancer Center were identified for inclusion (see Ethics Statement). Three preparation types were represented: aspirate smears prepared during rapid on‐site evaluation (ROSE) and cytospins manually coverslipped with CoverSeal‐X mounting medium (Cancer Diagnostics Inc., Durham, North Carolina, United States) and Ultraslips 24 × 50 mm, no. 1 glass coverslips (Cancer Diagnostics Inc.), whereas cell‐block sections were coverslipped with Tissue‐Tek film via the Tissue‐Tek Automated Coverslipper (Sakura Finetek USA Inc., Torrance, California, United States), which does not require mounting media. All slides had been stored at room temperature and ranged in age from approximately 1 to 23 years based on collection date.

#### 3.1.2 Experimental Design

Freeze durations were selected to span a pragmatic range from the shortest exposures reported in the literature (1–2 min) [9, 12] to a maximum compatible with routine clinical workflow (15 min), with intermediate timepoints chosen to capture the transition between ineffective and potentially effective exposures.

#### 3.1.3 Decoverslipping Procedure

Slides were placed coverslip‐side up in the assigned freezer (−20°C or −80°C) for the designated duration and removed immediately. Decoverslipping was attempted using a disposable microtome or scalpel blade. No solvents or rescue interventions were employed. Observations, including resistance, mounting medium behaviour and glass damage, were recorded.

#### 3.1.4 Outcomes

The primary endpoint was decoverslipping success, assessed using a novel ordinal four‐point Decoverslip Success Score (0–3) that integrates ease of removal and preservation of underlying cellular material (Table 3). Scoring was performed by a blinded assessor following microscopic review of a predefined 600 mm^2^ region of interest encompassing the central slide area where cellular material is most densely deposited. Cellular retention was estimated by visual comparison of the decoverslipped slide, expressed as the proportion of cellular material remaining relative to baseline. Score definitions ranged from 3 (Success: coverslip releases with minimal force, ≥ 80% cellular retention, minimal residue) to 0 (Failure: coverslip irremovable or < 20% cellular retention); full definitions are provided in Table 3. The score was dichotomised into a secondary binary endpoint: Success (score = 3) versus Nonsuccess (score = 0–2). An expanded acceptable threshold (score ≥ 2) was also considered, recognising that 50%–79% cellular retention may be sufficient for some downstream applications. Coverslip removal percentage, region of interest cell retention and mountant residue (None, Minimal, Moderate, Maximal) were also recorded.

**TABLE 3:** Decoverslip success score: A four‐point ordinal scoring system integrating ease of coverslip removal and preservation of underlying cellular material.

Score	Descriptor	Ease of removal	ROI cell retention	Mountant residue
**3**	**Success**	Coverslip releases with minimal force; no rescue steps required	≥ 80% with preserved architecture	Minimal
**2**	**Acceptable**	Moderate force required; minor focal lifting or tearing	50%–79%	Minimal to moderate; manageable with standard cleanup
**1**	**Poor**	Difficult removal with widespread lifting or cracking	20%–49%	Significant
**0**	**Failure**	Coverslip cannot be removed without major tissue damage, or cannot be removed at all	< 20%	Not applicable

*Note:* Cellular retention was assessed by visual comparison of the decoverslipped slide with a preprocedure image. The score was dichotomised into Success (score = 3) versus Nonsuccess (score = 0–2) for primary analysis, with an expanded acceptable threshold (≥ 2) also considered.

Abbreviation: ROI, region of interest (predefined 600 mm^2^ area encompassing the central slide region).

#### 3.1.5 Statistical Considerations

Given the exploratory nature of the study, the modest sample size within each experimental condition, and the high degree of intra‐case variability observed, formal inferential testing was not performed. Results are reported descriptively with case‐level and slide‐level detail.

### 3.2 Results

Systematic evaluation across slide preparation types revealed marked differences in decoverslipping performance (See Figure 1A,B and Table 4). Cell block section preparations, characterised by thinner mountant layers and more flexible film coverslips, were reliably decoverslipped under both temperature conditions and at all exposure durations. Aspirate smears, by contrast, exhibited substantial and consistent resistance to all freezing‐based approaches regardless of temperature, duration, stain type or blade type, and no single experimental condition achieved reliable and successful removal for this preparation type. No consistent difference in decoverslipping performance was observed between Diff‐Quik–stained (air‐dried) and Papanicolaou‐stained (alcohol‐fixed) slides (Table 4).

**FIGURE 1:**
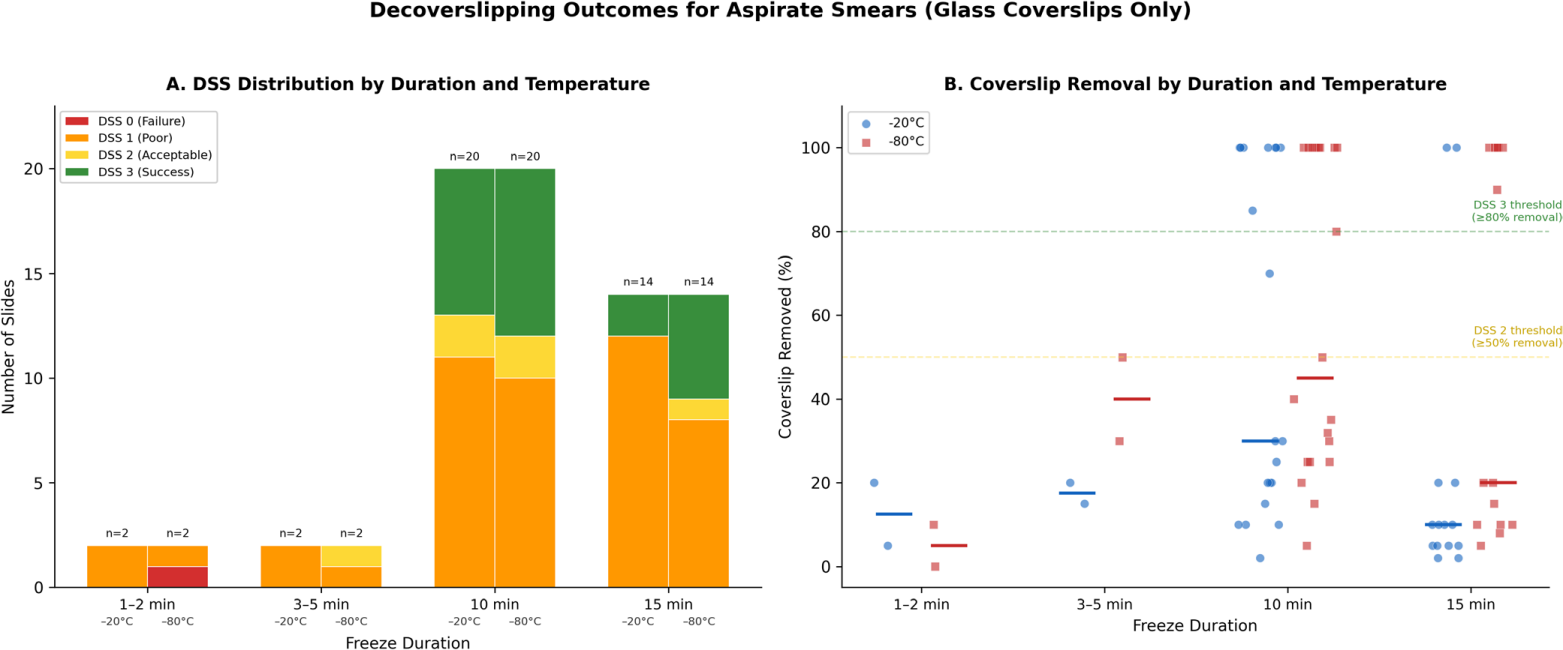
Decoverslipping outcomes for aspirate smears with glass coverslips (*n* = 76 slides; film coverslip cases C20 and C32 excluded). (A) Distribution of Decoverslip Success Scores (Decoverslip Success Score 0–3) stratified by freeze duration and temperature. Stacked bars show the number of slides achieving each score within each experimental condition. Decoverslip Success Score 3 (Success, green) indicates complete coverslip removal with ≥ 80% cellular material retention; Decoverslip Success Score 2 (Acceptable, yellow) indicates 50%–79% retention; Decoverslip Success Score 1 (Poor, orange) indicates 20%–49% retention; Decoverslip Success Score 0 (Failure, red) indicates < 20% retention or inability to remove the coverslip. Left bars within each duration group represent −20°C; right bars represent −80°C. (B) Individual slide‐level coverslip removal percentages by freeze duration and temperature. Each point represents a single slide (blue circles, −20°C; red squares, −80°C); horizontal bars indicate median values. Dashed lines denote the Decoverslip Success Score 3 threshold (≥ 80% removal, green) and Decoverslip Success Score 2 threshold (≥ 50% removal, yellow). Jitter is applied for visualisation. Note the marked intracondition variability, particularly at 10‐and 15‐min durations, reflecting the predominant influence of slide‐specific factors over experimental conditions.

**TABLE 4:** Case‐level allocation and slide‐level decoverslipping outcomes.

Case ID	Year	Slide	Stain	Freeze temp (°C)	Freeze duration	ROI cell retention (%)	Mountant residue	Coverslip removed (%)	Decoverslip score (0–3)	Dichotomised success
**C01**	2015	S1	Pap	−20	1–2 min	100	Minimal	20%	1	Nonsuccess
		S2	Pap	−80	1–2 min	100	Minimal	0%	0	Nonsuccess
**C02**	2015	S1	Pap	−20	3–5 min	100	Minimal	20%	1	Nonsuccess
		S2	Pap	−80	3–5 min	100	Minimal	30%	1	Nonsuccess
**C03**	2015	S1	DQ	−20	1–2 min	100	Minimal	5%	1	Nonsuccess
		S2	Pap	−80	1–2 min	100	Minimal	10%	1	Nonsuccess
**C04**	2015	S1	DQ	−20	3–5 min	100	Minimal	15%	1	Nonsuccess
		S2	DQ	−80	3–5 min	100	Minimal	50%	2	Nonsuccess
**C05**	2002	S1	Pap	−20	10 min	100	Minimal	100%	3	**Success**
		S2	Pap	−80	10 min	100	Minimal	100%	3	**Success**
**C06**	2015	S1	Pap	−20	10 min	100	Minimal	20%	1	Nonsuccess
		S2	Pap	−80	10 min	100	Minimal	30%	1	Nonsuccess
**C07**	2016	S1	Pap	−20	10 min	100	Minimal	20%	1	Nonsuccess
		S2	Pap	−80	10 min	100	Minimal	100%	3	**Success**
**C08**	2016	S1	Pap	−20	10 min	100	Minimal	30%	1	Nonsuccess
		S2	Pap	−80	10 min	100	Minimal	100%	3	**Success**
**C09**	2016	S1	Pap	−20	10 min	100	Minimal	10%	1	Nonsuccess
		S2	Pap	−80	10 min	100	Minimal	40%	1	Nonsuccess
**C10**	2015	S1	Pap	−20	10 min	100	Minimal	10%	1	Nonsuccess
		S2	Pap	−80	10 min	100	Minimal	50%**	2	Nonsuccess
**C11**	2024	S1	Pap	−20	10 min	100	Minimal	20%	1	Nonsuccess
		S2	Pap	−80	10 min	100	Minimal	35%	1	Nonsuccess
**C12**	2024	S1	Pap	−20	10 min	100	Minimal	30%	1	Nonsuccess
		S2	Pap	−80	10 min	100	Minimal	15%	1	Nonsuccess
**C13**	2016	S1	Pap	−20	10 min	100	Minimal	25%	1	Nonsuccess
		S2	Pap	−80	10 min	100	Minimal	5%	1	Nonsuccess
**C14**	2016	S1	Pap	−20	10 min	100	Minimal	2%	1	Nonsuccess
		S2	Pap	−80	10 min	100	Minimal	20%	1	Nonsuccess
**C15**	2016	S1	Pap	−20	10 min	100	Minimal	15%	1	Nonsuccess
		S2	Pap	−80	10 min	100	Minimal	25%	1	Nonsuccess
**C16**	2017	S1	Pap	−20	10 min	100	Moderate	100%	3	**Success**
		S2	Pap	−80	10 min	100	Minimal	32%	1	Nonsuccess
**C17**	2019	S1	Pap	−20	10 min	100	Minimal	100%	3	**Success**
		S2	DQ	−80	10 min	100	Minimal	100%	3	**Success**
**C18**	2016	S1	Pap CS	−20	10 min	100	Moderate	100%	3	**Success**
		S2	Pap CS	−80	10 min	100	Moderate	25%	1	Nonsuccess
**C19**	2016	S1	Pap	−20	10 min	80	Maximal	100%	3	**Success**
		S2	DQ	−80	10 min	80	Maximal	100%	3	**Success**
**C20**	2015	S1	HE CB*	−20	10 min	90	Maximal	100%	3	**Success**
		S2	HE CB*	−80	10 min	40	Minimal	70%	2	Nonsuccess
**C21**	2015	S1	Pap	−20	10 min	100	Minimal	10%	1	Nonsuccess
		S2	Pap	−80	10 min	100	Minimal	25%	1	Nonsuccess
**C22**	2002	S1	Pap CS	−20	10 min	70	Minimal	100%	3	**Success**
		S2	Pap CS	−80	10 min	50	Minimal	100%	3	**Success**
**C23**	2014	S1	Pap	−20	10 min	100	Minimal	85%	2	Nonsuccess
		S2	Pap	−80	10 min	100	Minimal	100%	3	**Success**
**C24**	2021	S1	DQ	−20	10 min	100	Minimal	70%	2	Nonsuccess
		S2	DQ	−80	10 min	100	Minimal	80%	2	Nonsuccess
**C25**	2021	S1	Pap	−20	10 min	100	Minimal	100%	3	**Success**
		S2	DQ	−80	10 min	100	Minimal	100%	3	**Success**
**C26**	2021	S1	Pap	−20	15 min	100	Minimal	100%	3	**Success**
		S2	Pap	−80	15 min	100	Minimal	100%	3	**Success**
**C27**	2021	S1	Pap	−20	15 min	100	Minimal	100%	3	**Success**
		S2	DQ	−80	15 min	100	Minimal	100%	3	**Success**
**C28**	2024	S1	Pap	−20	15 min	100	Minimal	10%	1	Nonsuccess
		S2	DQ	−80	15 min	100	Minimal	10%	1	Nonsuccess
**C29**	2024	S1	DQ	−20	15 min	100	Minimal	20%	1	Nonsuccess
		S2	Pap	−80	15 min	100	Minimal	20%	1	Nonsuccess
**C30**	2023	S1	Pap	−20	15 min	100	Minimal	10%	1	Nonsuccess
		S2	Pap	−80	15 min	100	Minimal	10%	1	Nonsuccess
**C31**	2015	S1	Pap	−20	15 min	100	Minimal	20%	1	Nonsuccess
		S2	DQ	−80	15 min	100	Minimal	100%	3	**Success**
**C32**	2015	S1	HE CB*	−20	15 min	40	Minimal	90%	2	Nonsuccess
		S2	HE CB*	−80	15 min	0	Minimal	100%	3	**Success**
**C33**	2022	S1	Pap	−20	15 min	100	Minimal	5%	1	Nonsuccess
		S2	DQ	−80	15 min	100	Minimal	8%	1	Nonsuccess
**C34**	2022	S1	Pap	−20	15 min	100	Minimal	5%	1	Nonsuccess
		S2	DQ	−80	15 min	100	Minimal	100%	3	**Success**
**C35**	2021	S1	Pap	−20	15 min	100	Minimal	2%	1	Nonsuccess
		S2	Pap	−80	15 min	100	Minimal	20%	1	Nonsuccess
**C36**	2021	S1	Pap	−20	15 min	100	Minimal	10%	1	Nonsuccess
		S2	Pap	−80	15 min	100	Minimal	15%	1	Nonsuccess
**C37**	2024	S1	DQ	−20	15 min	100	Minimal	5%	1	Nonsuccess
		S2	DQ	−80	15 min	100	Minimal	10%	1	Nonsuccess
**C38**	2024	S1	DQ	−20	15 min	100	Minimal	10%	1	Nonsuccess
		S2	DQ	−80	15 min	100	Minimal	100%	3	**Success**
**C39**	2024	S1	Pap	−20	15 min	100	Minimal	5%	1	Nonsuccess
		S2	Pap	−80	15 min	100	Minimal	90%	2	Nonsuccess
**C40**	2024	S1	DQ	−20	15 min	100	Minimal	2%	1	Nonsuccess
		S2	Pap	−80	15 min	100	Minimal	5%	1	Nonsuccess

*Note:* ROI Cell Retention: estimated percentage of cellular material preserved within a predefined 600 mm^2^ region of interest. Mountant Residue: graded as None, Minimal, Moderate or Maximal. Decoverslip Score: 0 = failed removal; 1 = < 50% coverslip removed; 2 = ≥ 50% but incomplete; 3 = complete removal without tissue damage. Dichotomised Success: Score 3 = Success; Scores 0–2 = Nonsuccess – indicates data not recorded.

Abbreviations: CB, cell block; CS, cytospin; DQ, Diff‐Quik; HE, haematoxylin and eosin; Pap, Papanicolaou stain.

* Indicates a Plastic Coverslip was Identified, Precluding Standard Freeze–Fracture Decoverslipping.

** Slide accidentally broken during procedure; result should be interpreted with caution.

#### 3.2.1 Aspirate Smear Outcomes

A total of 40 aspirate smear cases (80 slides) were evaluated across four freeze‐duration groups (1–2 min, 3–5 min, 10 min and 15 min) at two temperature conditions (−20°C and −80°C), with complete successful removal achieved in only 26 of 80 total slides (32.5%). Two cases (C20 and C32) were identified as cell block section slides bearing film coverslips (denoted * in Table 4) and were excluded from primary comparative analyses, yielding 38 evaluable aspirate smear cases (76 slides) for the main analysis. Among these 76 slides, complete coverslip removal (100%) was achieved in 22 (28.9%), while the majority of slides yielded less than 50% removal regardless of temperature condition or freeze duration. When the expanded acceptable success threshold (Decoverslip Success Score ≥ 2) was applied, 28 of 76 slides (36.8%) achieved at least acceptable outcomes, compared with 22 (28.9%) at the stringent score = 3 threshold, indicating that an additional six slides retained 50%–79% of cellular material, which is a yield that may be sufficient for selected downstream ancillary testing applications.

#### 3.2.2 Effect of Freeze Duration

Freeze duration was the most influential experimental variable. Short exposures of 1–2 min (4 slides, 2 cases) and 3–5 min (4 slides, 2 cases) were uniformly ineffective; no slide achieved a Decoverslip Success Score above 2. It should be noted that these short‐duration groups contain very small sample sizes, as the study design was adapted sequentially: when initial short freeze durations yielded no successful outcomes, subsequent cases were allocated to longer exposure durations. Extension to 10 min (40 evaluable slides) produced the highest success rate, with 15 of 40 slides (37.5%) achieving complete removal, though performance remained highly variable. Further extension to 15 min (28 evaluable slides) paradoxically yielded a lower success rate of 7 of 28 slides (25.0%), suggesting diminishing and unpredictable returns beyond 10 min for this preparation type.

#### 3.2.3 Effect of Freeze Temperature

Neither −20°C nor −80°C demonstrated a consistent, clinically actionable advantage across the full dataset; however, a directional trend favouring −80°C was apparent, particularly at longer exposure durations. At 10 min, −80°C yielded equal or greater coverslip removal in 15 of 19 case‐level comparisons. At 15 min, −80°C produced equal or greater removal in all 14 evaluable comparisons, with particularly striking differences in individual cases (e.g., C34: 100% vs. 5%; C38: 100% vs. 10%). Despite this directional consistency, the absolute success rate remained low, and neither temperature condition overcame the fundamental resistance of this preparation type.

#### 3.2.4 Intra‐Case Variability

Marked within‐case variability was observed between paired slides processed under the same freeze duration but at different temperatures. Eight cases exhibited differences in coverslip removal exceeding 65 percentage points, with the most extreme discordance reaching 95 percentage points (C34: 5% vs. 100%). An additional contributor to intra‐case variation may be brief post‐coverslipping drying with heating, a step applied to some aspirate slides following the rapid on‐site evaluation call and to some cytospin preparations.

#### 3.2.5 Influence of Slide Age

Cases in this cohort spanned collection years from 2002 to 2024, representing slide ages of approximately 1–23 years at the time of the study. To explore whether slide age influenced decoverslipping performance, outcomes were examined by collection year across age brackets. Interpretation of these data requires the caveat that freeze‐duration assignment was not balanced across collection years. Apparent age effects are therefore partly confounded by duration allocation.

A directional association between slide age and decoverslipping success was observed: the oldest cases (2002, age 23 years) achieved 100% success across all four slides, intermediate‐age cases showed variable success (31%–50%) and the most recent cases (2024, age 1 year) yielded only 6.2% success. This pattern persisted when analysis was restricted to the 10‐min group to reduce confounding by duration allocation, though the relationship was nonlinear. However, this association was accompanied by a trade‐off: the oldest cases demonstrated reduced cellular retention (50%–70%), whereas all slides from 2014 to 2024 retained 100% (with one exception).

#### 3.2.6 Film Coverslip Cases

Two cell block cases (C20 and C32) bearing film coverslips were excluded from primary direct smear analyses. Both achieved complete or near‐complete physical removal (70%–100%) but with variable cellular preservation (0%–100% retention) and frequent mountant residue, consistent with film coverslips being mechanically amenable to peeling but less reliable for cellular integrity.

#### 3.2.7 Region of Interest Cell Retention and Mountant Residue

Region of interest cell retention was 100% in the majority of evaluable glass‐coverslip slides, with notable exceptions in the oldest cases (C22: 70%–50%). Film coverslip cases showed more variable retention (0%–100%). Mountant residue was minimal in the overwhelming majority of glass‐coverslip cases; moderate residue was recorded for C16‐S1 and both slides of C18, and maximal residue was recorded for both slides of C19. Among the film coverslip cases, maximal residue was observed for C20‐S1.

## 4 Discussion

The interaction between a specific mounting medium and the chosen decoverslipping method, rather than either variable in isolation, governs both the feasibility of coverslip removal and the quality of recovered analytes [5, 6, 9]. Published freezing‐based methods generally outperform solvent‐only approaches in speed and cellular preservation; however, reported outcomes vary considerably with mounting‐medium formulation and specimen type (Table 1). Dejmek et al. [6] reported superior DNA recovery with xylene‐free mounting media (EcoMount (Biocare Medical, Pacheco, California, United States), Histo‐Clear (National Diagnostics, Atlanta, Georgia, United States)). Ramesh et al. [5] found that xylene produced higher‐quality DNA than −80°C freezing, and Dinesh et al. [9] demonstrated that freezing was fastest for decoverslipping DPX‐mounted archival slides but did not directly compare nucleic acid outcomes. Our aspirate smears, prepared with CoverSeal‐X, a xylene‐based resinous polymer formulation comprising ethylbenzene and butylated hydroxytoluene, were consistently refractory to freezing approaches (28.9% slide‐level success), in marked contrast to the effective freezer‐based removal of DPX‐mounted slides reported by Dinesh et al. [9]. The rigidity and brittleness of glass coverslips, which are less responsive to differential thermal contraction than film alternatives, likely compounded this resistance. This divergence confirms mounting medium composition and coverslip material as the dominant preanalytical variables governing decoverslipping feasibility.

Among the experimental variables evaluated, freeze duration exerted the greatest influence on decoverslipping outcome. Exposures of 5 min or less were uniformly ineffective, consistent with prior reports that brief cooling alone is insufficient to embrittle thick resinous mounting media [9, 11]. Extension to 10 min produced the highest per‐slide success rate (37.5%), suggesting a threshold at which differential thermal contraction between the glass coverslip and underlying resin becomes sufficient to disrupt adhesion. The paradoxical decline at 15 min (25.0%) may reflect thermal equilibration, diminishing the contraction mismatch that facilitates separation. A directional advantage for −80°C over −20°C was apparent at both 10‐ and 15‐min exposures, with −80°C yielding equal or greater removal in 29 of 33 case‐level comparisons, including striking individual differences (e.g., C34: 100% vs. 5%). The greater thermal differential at −80°C likely produces more rapid and complete embrittlement; nevertheless, neither temperature condition reliably overcame the resistance conferred by thick CoverSeal‐X mounting medium and rigid glass coverslips.

Marked intra‐case variability between paired slides, with discordance exceeding 65 percentage points in eight cases (C07, C08, C16, C18, C31, C34, C38 and C39), indicates that local, slide‐specific factors such as mountant layer thickness and post‐coverslipping heating dominate outcome independently of any controlled experimental condition. This degree of unpredictability suggests that no single freezing protocol can be expected to yield consistent results for aspirate smears, and that slide‐by‐slide assessment will be necessary even under optimised conditions. By contrast, the fixation method did not appreciably influence adhesion; no consistent difference was observed between Diff‐Quik‐stained (air‐dried) and Papanicolaou‐stained (alcohol‐fixed) preparations, indicating that this variable need not factor into method selection. When the expanded acceptable threshold (Decoverslip Success Score ≥ 2) was applied, an additional 6 slides (36.8% vs. 28.9%) achieved outcomes potentially sufficient for targeted molecular assays that tolerate partial cellular input. Mountant residue was minimal in most cases but moderate to maximal in C16, C18 and C19, warranting consideration of post‐decoverslipping cleanup protocols.

Slide age emerged as an additional variable of interest: the oldest specimens (23 years old) achieved uniformly successful removal, whilst the most recent (1 year old) performed poorly (6.2% slide‐level success). However, successful removal of aged slides was accompanied by reduced cellular retention (notably C22, with 50%–70%), highlighting a trade‐off between decreased adhesion and progressive cellular degradation that warrants prospective investigation. Progressive polymerisation and oxidative degradation of the mounting medium likely render the resin more brittle but concurrently compromise cellular integrity. Prospective standardisation of coverslip materials and mounting formulations, rather than retrospective adaptation, may represent the most sustainable approach to ensuring downstream ancillary testing compatibility.

Glass coverslips are rigid and brittle; when bonded by thick, polymerised mounting medium, they resist mechanical removal and are prone to shattering [15]. Film coverslips, by contrast, are flexible and permit peeling rather than fracture, and perform well on preparations with uniform surface profiles such as formalin‐fixed paraffin‐embedded sections and liquid‐based cytology preparations [16, 17]. However, automated film coverslipping is designed for high‐throughput histology workflows and is not readily applicable to direct aspirate smears, which are typically prepared at the bedside and manually coverslipped with glass, particularly in the context of ROSE, where immediate slide preparation and interpretation preclude incorporation into automated coverslipping platforms. Whether film coverslips perform equivalently on direct aspirate smears, which have markedly uneven cell distribution and variable smear thickness, has not been systematically evaluated; the irregular topography of direct smears could plausibly compromise film adherence or introduce optical distortion.

For aspirate smears resistant to freeze‐based methods, controlled heat‐assisted decoverslipping represents a logical alternative with precedent in cytology workflows. Hot‐plate methods employing 120°C for up to 30 s (freshly prepared slides) or up to 170°C for approximately 2 min (older slides) have been described [4]; however, the impact of these temperatures on protein and nucleic acid integrity has not been systematically characterised. Traditional prolonged xylene soaking (typically 2–4 h or overnight) remains the most established fallback for refractory preparations [4, 5, 6], though it does not eliminate organic solvent exposure. Defining the nucleic acid quality thresholds achievable with each approach constitutes an important evidence gap that must be addressed before heat‐assisted methods can be responsibly adopted in precision oncology pipelines.

This study has several limitations. The Decoverslip Success Score is a novel single‐assessor metric without inter‐rater reliability data; importantly, it evaluates morphological preservation rather than protein or nucleic acid integrity, which would require separate assessment. This is notable given that the primary rationale for decoverslipping is to enable downstream ancillary testing, including immunohistochemistry, fluorescence in situ hybridisation and molecular assays. Sample sizes within the short‐duration freeze groups (1–2 min and 3–5 min) were very small (4 slides each), and the sequential allocation design means that findings for these durations are preliminary. Slide age was partly confounded by freeze‐duration assignment, precluding definitive conclusions about the independent effect of specimen age. Lastly, the study was conducted at a single institution using a single mounting medium formulation (CoverSeal‐X), and results may not generalise to other mounting media, coverslip types or institutional workflows.

## 5 Practical Recommendations

Based on the literature review and our institutional experience, the following practical recommendations are offered for laboratories considering decoverslipping of archival cytology slides for downstream ancillary testing:

1. Assess slide preparation type before selecting a decoverslipping method. Slide preparations with thin mountant layers and film coverslips are generally amenable to freezer‐based removal at −20°C or −80°C for 10 min. Aspirate smears prepared with thick resin‐based mounting media and rigid glass coverslips are likely to be refractory to cold‐based approaches, and alternative strategies such as prolonged xylene soaking or controlled heat‐assisted removal may be considered as first‐line options for this preparation type.
2. Record the mounting medium type and coverslip material at the time of slide preparation. This information is rarely documented in current practice, but it is a critical preanalytical variable that governs decoverslipping feasibility. Standardised recording of these details in laboratory information systems would facilitate informed method selection when retrospective ancillary testing is required.
3. When freezer‐based decoverslipping is attempted, an initial exposure of 10 min at −80°C is recommended as the starting point. Shorter durations (≤ 5 min) are unlikely to be effective for most preparation types, and durations beyond 10 min do not reliably improve success rates.
4. Consider prospective alignment of coverslipping practices with anticipated downstream molecular needs. Where future ancillary testing is likely, laboratories should consider using thinner mounting medium applications or film coverslips, which are more amenable to mechanical removal, as they are more flexible and less prone to shattering.
5. Exercise appropriate safety precautions during mechanical decoverslipping. Blade‐assisted removal of glass coverslips carries a risk of glass chipping and should be performed with eye protection and cut‐resistant gloves in a designated workspace.

## 6 Conclusion

Decoverslipping is an underestimated determinant of ancillary testing feasibility from archival cytology slides. While freezing‐based methods may be effective for certain preparations, aspirate smears prepared with thick xylene‐based mounting media and rigid glass coverslips are consistently refractory, with complete removal achieved in only approximately one third of evaluable slides. Large intra‐case variability between paired slides confirms that slide‐specific factors, principally mounting‐medium composition and coverslip material, rather than freezing protocol, are the dominant drivers of outcome. These findings stress the need to prospectively align coverslipping materials and practices with downstream ancillary testing requirements. Future work should characterise the impact of heat‐assisted decoverslipping on nucleic acid integrity and evaluate alternative mounting strategies optimised for contemporary precision oncology workflows.

## Author Contributions

Nyein Nyein Htun: investigation, data curation, writing – review and editing. Benjamin Morris: conceptualisation, writing – review and editing. Sinchita Roy‐Chowdhuri: conceptualisation, writing – review and editing. Gloria Hopkins Sura: conceptualisation, methodology, investigation, formal analysis, writing – original draft, writing – review and editing, supervision.

## Funding

The authors have nothing to report.

## Disclosure

Mina Desai Early Career Investigator Award Nomination: Nyein Nyein Htun is nominated for the Mina Desai Early Career Investigator Award. Dr. Htun is currently in a training position in Cytopathology at The University of Texas MD Anderson Cancer Center and contributed extensively to this work, including investigation, data curation and critical review of the manuscript.

## Ethics Statement

This study was conducted using archival, de‐identified cytology slides derived from clinical cases at The University of Texas MD Anderson Cancer Center. The protocol was reviewed by the Institutional Review Board, which granted a waiver of informed consent due to the retrospective nature of the study and the use of existing pathological materials without patient contact, intervention or identifiable patient information. All procedures were performed in accordance with institutional guidelines and ethical standards, including the principles of the Declaration of Helsinki.

## Consent

All patients whose archival cytology specimens were included in this study had previously signed an informed consent document at The University of Texas MD Anderson Cancer Center acknowledging that their data and/or biological samples may be used by MD Anderson researchers for future research purposes and may be shared with other investigators. All information and specimens used in this study were de‐identified prior to analysis, and no individually identifiable patient information was accessed or reported at any stage of the investigation.

## Conflicts of Interest

The authors declare no conflicts of interest.

## Data Availability

All data generated during this study are included in this published article. The complete slide‐level dataset is presented in Table 4.
